# Altered Expression of Endoplasmic Reticulum Stress Associated Genes in Hippocampus of Learned Helpless Rats: Relevance to Depression Pathophysiology

**DOI:** 10.3389/fphar.2015.00319

**Published:** 2016-01-12

**Authors:** Matthew A. Timberlake, Yogesh Dwivedi

**Affiliations:** Department of Psychiatry and Behavioral Neurobiology, The University of Alabama at Birmingham School of Medicine, BirminghamAL, USA

**Keywords:** learned helplessness, hippocampus, depression, ER stress, UPR system, gene expression

## Abstract

The unfolded protein response (UPR) is an evolutionarily conserved defensive mechanism that is used by cells to correct misfolded proteins that accumulate in the endoplasmic reticulum. These proteins are misfolded as a result of physical stress on a cell and initiate a host of downstream effects that govern processes ranging from inflammation to apoptosis. To examine whether UPR system plays a role in depression, we examined the expression of genes that are part of the three different pathways for UPR activation, namely GRP78, GRP94, ATF6, XBP-1, ATF4, and CHOP using an animal model system that distinguishes vulnerability (learned helpless, LH) from resistance (non-learned helpless, NLH) to develop depression. Rats were exposed to inescapable shock on days 1 and 7 and were tested for escape latency on day 14. Rats not given shock but tested for escape latency were used as tested control (TC). Plasma corticosterone (CORT) levels were measured. Expression levels of various UPR associated genes were determined in hippocampus using qPCR. We found that the CORT level was higher in LH rats compared with TC and NLH rats. Expression of GRP78, GRP94, ATF6, and XBP-1 were significantly upregulated in LH rats compared with TC or NLH rats, whereas NLH rats did not show such changes. Expression levels of ATF4 and CHOP showed trends toward upregulation but were not significantly altered in LH or NLH group. Our data show strong evidence of altered UPR system in depressed rats, which could be associated with development of depressive behavior.

## Introduction

Major depressive disorder (MDD) is a debilitating psychiatric disorder that affects about 10% of all adults in the United States. Symptoms of MDD include sadness and bereavement well after a depressive source is removed. In addition, MDD subjects show irritability, change in sleep/wake cycles, change in appetite and corresponding change in weight, focus, ability to complete tasks, sexual drive, and finding pleasure or reward in once-pleasurable activities (Belmaker and Agam, 2008). In the past decade several studies have focused on examining the etiological factors associated with depression, the exact molecular mechanism associated with this disorder is not clearly understood.

Several lines of investigation suggest that depressed brain is associated with structural abnormalities. These include studies in human postmortem brain (Aganova and Uranova, 1990; Ongur et al., 1998; Rajkowska, 2000, 2002; Rosoklija et al., 2000; Cotter et al., 2001, 2002; Miguel-Hidalgo and Rajkowska, 2002), magnetic resonance imaging (MRI) in depressed patients (Drevets et al., 1998; Fossati et al., 2004), and in animal models of stress and depression (McEwen, 2000). One well-documented phenomenon is the shrinkage and atrophy of hippocampus as shown in MRI and postmortem brain tissues in patients with MDD (Sala et al., 2004; Frodl et al., 2006). This phenomenon has been noted by many researchers but there have only been a few proposed mechanisms. Among other prevailing theories include the hyperactive hypothalamic-pituitary-adrenal axis (HPA-axis; Varghese and Brown, 2001) and growth factor dysregulation along with intracellular cellular cascade disruption (Dwivedi and Pandey, 2008; Dwivedi, 2009). Cellular stress has also been linked to many of the symptoms of depression (Lotrich, 2012). Cellular stress can be defined as the cell’s response to stressors including environmental, physical or any other stimulus that causes an excessive deviation from homeostatic norm (Welch, 1993). Within the cell, one particular organelle that shows an adaptive response to cellular stress is the endoplasmic reticulum (ER). Under normal conditions, the ER is responsible for the folding and trafficking of proteins from the intracellular to the extracellular membrane. Under stressful conditions, various internal cellular homeostatic norms can be disrupted, e.g., the levels of available ATP, which can lead to improper protein folding and thus functional loss (Kaufman, 2002). In response to this misfolding, the collective series of events known as the unfolded protein response (UPR) occurs. The UPR is a response where three basic steps are followed: (1) refold/correct misfolded proteins, (2) degrade/remove misfolded proteins when refolding/correcting is being overwhelmed, and finally (3) signal the cell to initiate apoptotic pathways (Kaufman, 2002). Xbox Binding Protein 1 (XBP1), Activating Transcription Factor 4 and 6 (ATF4 and ATF6), C/EBP Homologous Protein (CHOP), DNA-Damage-Inducible Transcript (DDIT3), and the chaperones Heat Shock 70 kDa Protein 5/Glucose Regulated protein 78 kD (GRP78) and Heat Shock Protein 90 kDa Beta/94 KDa Glucose-Regulated Protein (GRP94) are the key molecules that participate in such events.

There have been some studies that point to the role of UPR in mood disorders. For example, Hayashi et al. (2009) reported impaired ER stress response in lymphoblastoid cell lines derived from bipolar patients. A genetic association between bipolar disorder and HSP90B1 has also been found (Kakiuchi et al., 2007). A polymorphism (–116C→G) in the promoter region of XBP1, affecting the putative binding site of XBP1, was found to be significantly more common in Japanese bipolar patients and overtransmitted to affected offspring in trio samples of the NIMH Bipolar Disorder Genetics Initiative (Kakiuchi et al., 2003). In MDD patients, Grunebaum et al. (2009) reported significant genetic association of XBP1 with cortisol levels. More recently, Nevell et al. (2014) found persistent activation of the ER stress response in peripheral tissues of MDD patients. A recent study in rats also suggests that ER stress is involved in restraint stress-induced hippocampal apoptosis and cognitive impairments (Zhang et al., 2014). These studies provide some evidence of abnormal ER response in mood disorders; however, whether consequence of UPR system is associated with resiliency or resistant to develop depression is not clearly understood.

In the present study, we used the learned helpless (LH) model of depression that holds the distinct advantage of dichotomizing the cohort into a resilient and susceptible-to-depression group. This is considered one of the most valid and reliable animal models of depression. It represents the theoretical basis of the origin and development of depression, as this model is a combination of cognitive and neurovegetative abnormalities and genetic susceptibility (Jesberger and Richardson, 1985; Vollmayr and Henn, 2001; Nestler et al., 2002). Since regulation of key genes associated with the UPR system is essential in its activation or adaptive response, we examined the expression of critical transcription factors as well as chaperone proteins XBP1, ATF4, ATF6, CHOP, GRP7, and GRP94 in hippocampus of susceptible and resilient rats. We hypothesize that elements of the UPR hold the key to understanding the differences in resilient and susceptible rats and that the findings can have a significant impact in understanding the molecular pathophysiology of depression.

## Materials and Methods

### Animals

Male Holtzman rats (350–375 g body weight) were obtained from Harlan Sprague-Dawley Laboratories (Indianapolis, IN, USA) and were housed in individual cages (3/cage) under standard laboratory conditions (temperature 21 ± 1°C, humidity 55 ± 5%, 12-h light/dark cycle). All rats received *ad libitum* food and water. Animals were housed for 1 week prior to the start of experiment. All experiments were performed between 08:00 and 10:00 (under light cycle). Experimental procedures were approved by the Animal Care Committee of the University of Alabama at Birmingham. The animals were divided in three groups as follows: tested control (TC; *n* = 8), non-learned helpless (NLH; *n* = 8), and LH (*n* = 5)

### Induction of LH Behavior

#### Behavioral Procedures

**FIGURE 1:**
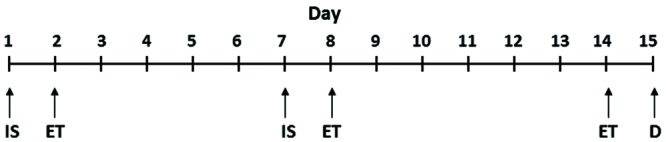
**Schematic representation of IS and ET paradigms.** IS, inescapable shock; ET, escape test; D, decapitation. The detailed procedure is provided in the “Materials and Methods” section.

The procedure for the induction of depressive behavior in rats was the same as described in our earlier publications (Dwivedi et al., 2004; Smalheiser et al., 2011). LH induction (inescapable shock, IS) and escape test (ET) paradigms for the different groups of rats are provided in **Figure 1**. Rats were given IS on day 1 and tested for escape behavior on day 2; these animals were given another IS on day 7 and tested for escape behavior on day 8 and on day 14. All the animals were sacrificed 24 h after the last escape testing during light cycle.

#### Inescapable Shock Treatment

The rats were placed in Plexiglass tubes with the rat’s tail extending from the rear of the tube. ISs were delivered to tails by means of a computer-controlled constant current shock generator (Model ENV-410B, Med Associates, Lafayette, IN, USA). The IS consisted of 100 random shocks delivered for 5 s at the rate of 1.0 mA. The mean interval between shocks was of 60 s. The TC rats were placed in Plexiglass tubes but did not receive shock.

#### Shuttle Escape Testing

Escape testing was conducted in a shuttle box (70 cm × 20 cm × 20 cm; Med Associates, Lafayette, IN, USA). The shuttle box contained an electrified grid floor and was divided into two equal chambers with an arched doorway in the center (5 cm × 7 cm). Foot shocks were delivered through the grid floor by a shock generator (0.6 mA on a *VI* schedule; Model # ENV-413, Med Associates, Lafayette, IN, USA). The shuttle escape testing began with five trials (FR-1) during which a single crossing would terminate the shock. This was followed by 25 trials (FR-2) in which a rat had to cross from one side of the shuttle box to the other and come back to terminate the shock. Shocks were terminated automatically after 30 s if there was no response within that time. Shuttle escape latencies were recorded automatically by a computer attached to the generator and shuttle box.

Rats were divided into two groups based on the mean latency observed after FR-2: (1) those rats in which the mean latency was ≥20 s (termed LH) and (2) those in which the mean latency was <20 s (termed as NLH). In our study, we found that about 50% of all the rats tested became LH rats. The TC rats were confined to Plexiglass tubes but were not shocked.

### Tissue Collection

Twenty-four hours after the final ET, rats were decapitated and trunk blood was collected (08:00–10:00). Brains were removed and the hippocampi were dissected and flash frozen in liquid nitrogen. Tissues were stored at -80°C until they were analyzed.

### Measurement of Corticosterone (CORT) Levels

The trunk blood was collected at the time of decapitation (8:00–10:00). Serum was isolated and was stored at -80°C until the assays were performed. Serum CORT levels were measured by a radioimmunoassay kit (ICN Biomedical Inc., Cleveland, OH, USA).

### RNA Isolation

RNA was isolated using TRIzol^®^ (Life Technologies, USA) as described earlier (Smalheiser et al., 2011). RNA degradation was assessed using denaturing agarose gel electrophoresis and evaluating the 28S and 18S rRNA band integrity.

### qPCR Method

One microgram total RNA was reverse transcribed using M-MLV Reverse Transcriptase (Invitrogen, Grandsland, NY, USA) and oligo (dT) primer. The oligo dT primer annealing step was carried out at 5 μM concentration in presence of 1 mM dNTPs by incubating the reaction at 65°C for 5 min. The reaction was quenched by holding at 4°C for more than 2 min. The reaction was mixed with 1X first strand synthesis buffer, 0.01 mM DTT, 2 U of RNaseOut and 200 U of M-MLV Reverse Transcriptase and incubated at 37°C for 50 min. Finally the reaction was inactivated at 70°C. Relative abundance of transcripts were measured with a quantitative real time PCR machine (Stratagen MxPro3005, La Jolla, CA, USA) using 1X EvaGreen qPCR mastermix (Applied Biological Material Inc., Canada) in combination with 0.8 μM each of gene specific forward and reverse primers. Forty-fold diluted raw cDNA was used as template for qPCR amplification using a thermal parameter of initial denaturation at 95°C for 10 min followed by a repeating 40 cycles of denaturation at 95°C for 10 s, primer annealing at 60°C for 15 s and an extension of amplicon at 72°C for 20 s. Possibility of primer dimer formation and secondary product amplification was ruled out by running a single cycle of EvaGreen specific dissociation curve analysis program with initial denaturation at 95°C for 1 min followed by annealing at 55°C for 30 s and repeat denaturation 95°C for 30 s. Relative gene expression level was quantified after normalization with GAPDH as reference gene and fold change value determined following Livak’s ^ΔΔ^*C*_t_ calculation method (Livak and Schmittgen, 2001). Data are presented as fold change.

Following are the primer sequences: GRP78 forward: 5′GCAGTTGCTCACGTGTCTTG/reverse: 5′TCCAAGGTGAACACACACCC; GRP94 forward: 5′AAACGGCAACTCTTCGG TCA/reverse: 5′TTAAGCTGAGGCGGAGCATC; ATF6 forward: 5′CGAGGGAGAGGT GTCTGTTTC/reverse: 5′GTCTTCACCTGGTCCA TGAGG; XBP-1, forward: 5′CCA CTTGGTACAGACCACTCC/reverse: 5′AGACACTA ATCAGCTGGGGG; ATF4 forward: 5′AAGGCAGATTCTCTCGCCAA/reverse: 5′TTCTTCCCCCTTGCCTTACG; CHOP forward: 5′AGGA/GAGAGAAACCGGTCCAA/reverse: 5′GGACACTGTCTCAAAGGCGA; GAPDH forward 5′CACTGAGCATCTCCCTC ACAA/reverse: 5′TGGTATTCGAGAGA AGGGAGG.

### Statistical Analysis

Statistical Package for the Social Sciences (SPSS) was used for all the data analysis. The data are represented as mean ± SD. TC, LH, and non-LH groups were compared using one-way ANOVA. *Post hoc* comparisons were calculated by Tukey’s method of multiple comparisons. Significance level was set at *p* < 0.05.

## Results

### Escape Latencies

**FIGURE 2:**
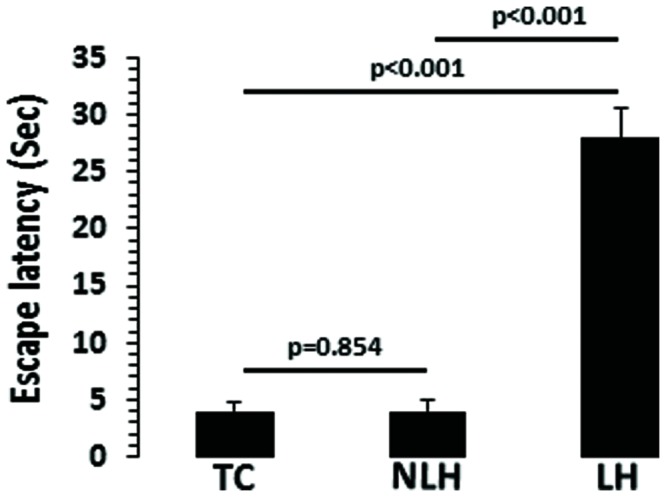
**Escape latencies in tested control (TC; *n* = 8), non-learned helpless (NLH; *n* = 8), and learned helpless (LH; *n* = 5) rats.** The group difference in escape latencies between TC, NLH, and LH rats is as follows: *df* = 2,18, *F* = 533.381, *p* < 0.001.

There was a significant difference in escape latency among TC, NLH, and LH rats (*df* = 2,18, *F* = 533.381, *p* < 0.001). No significant differences were noted between NLH and TC rats. The mean latencies are depicted in **Figure 2**. Individual group analysis revealed that LH group was significantly different from NLH or TC groups (*p* < 0.001). There was no significant difference between NLH and TC groups (*p* = 0.854).

### Corticosterone Levels

**FIGURE 3:**
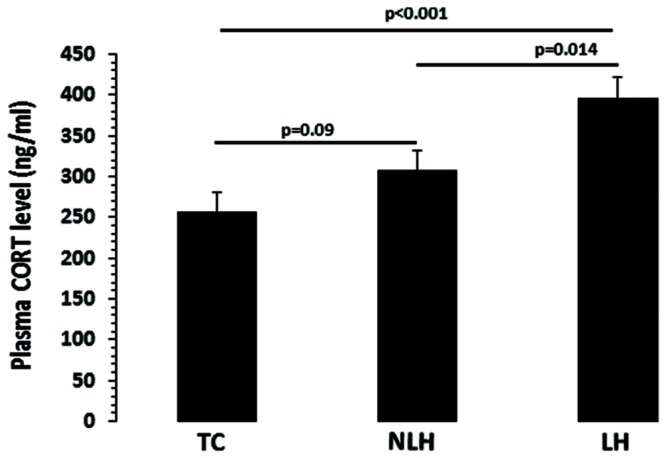
**Plasma corticosterone (CORT) levels in TC (*n* = 8), NLH (*n* = 8), and LH (*n* = 5) rats.** Data are the mean ± SEM. The differences in serum CORT levels between the TC, NLH, and LH groups are as follows: *df* = 2,18, *F* = 9.21, *p* = 0.002.

The differences in serum CORT levels between the TC, NLH, and LH groups were as follows: *df* = 2,18, *F* = 9.21, *p* = 0.002). As shown in **Figure 3**, the CORT levels were significantly higher in LH rats compared with TC rats (*p* < 0.001) and NLH rats (*p* = 0.014). No significant differences were noted between NLH and TC rats (*p* = 0.09).

### mRNA Expression Analysis

**FIGURE 4:**
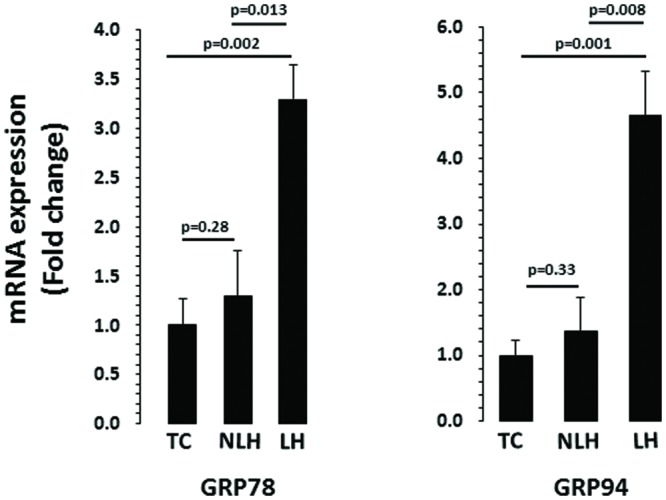
**mRNA expression of GRP78 and GRP94 in hippocampus of TC (*n* = 8), NLH (*n* = 8), and LH (*n* = 5) rats.** mRNA levels were determined by qRT-PCR using SyberGreen^®^. Data are the mean ± SEM. The group differences in the three groups are as follows: GRP78: *df* = 2,18, *F* = 7.068, *p* = 0.005; GRP94: *df* = 2,18, *F* = 7.717, *p* = 0.004.

Expression levels of various genes associated with UPR system were evaluated in hippocampus of LH, NLH, and TC rats. GAPDH was used as endogenous control. The expression level of GAPDH did not differ significantly among the three groups (*df* = 2,18, *F* = 0.44, *p* = 0.22). One-way ANOVA showed that GRP78 expression were significantly different between TC, NLH, and LH groups (*df* = 2,18; *F* = 7.06; *p* = 0.005). **Figure 4** shows that the expression of GRP78 was significantly upregulated in LH rats when compared with TC rats (*p* = 0.002) and NLH rats (*p* = 0.013). No differences between NLH and TC rats were observed (*p* = 0.28).

For chaperone GRP94, the overall group difference between the TC, NLH, and LH rats was: *df* = 2,18; *F* = 7.72; *p* = 0.004. mRNA level of GRP94 was significantly increased in LH rats compared with TC (*p* = 0.001) or NLH (*p* = 0.008) rats (**Figure 4**). No significant difference in the expression of GRP94 was found between NLH and TC (*p* = 0.33) rats.

**FIGURE 5:**
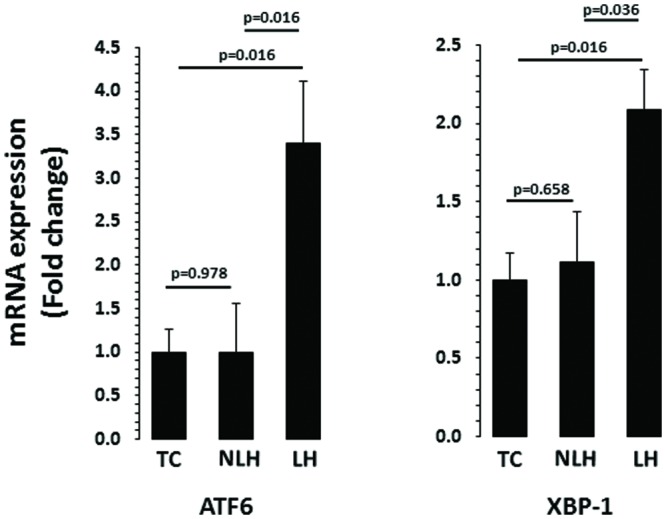
**mRNA expression of ATF6 and XBP-1 in hippocampus of TC (*n* = 8), NLH (*n* = 8), and LH (*n* = 5) rats.** mRNA levels were determined by qRT-PCR using SyberGreen^®^. Data are the mean ± SEM. The group differences in the three groups are as follows: ATF6: *df* = 2,18, *F* = 4.370, *p* = 0.028; XBP-1: *df* = 2,18, *F* = 3.862, *p* = 0.04.

mRNA levels of ATF6 and XBP-1 are depicted in **Figure 5**. There was a significant differences in the expression of chaperone ATF6 between TC, NLH, and LH groups (*df =* 2,18; *F* = 4.370; *p* = 0.028). mRNA level of ATF6 was significantly higher in the LH group compared with TC (*p* = 0.016) and NLH (*p* = 0.016) groups. No significant difference was noted between NLH and TC (*p* = 0.978) groups. As with ATF6, ANOVA showed significant difference between TC, NLH, and LH groups for XBP-1 (*df* = 2,18; *F* = 3.86; *p* = 0.04). The gene expression of XBP-1 was significantly elevated in the LH group compared with TC (*p* = 0.016) and NLH (*p* = 0.036) groups. There was no significant difference between NLH and TC (*p* = 0.658; **Figure 5**).

**FIGURE 6:**
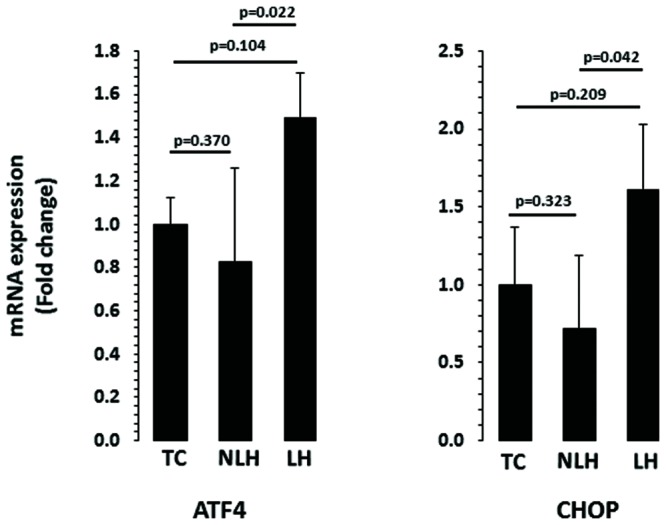
**mRNA expression of ATF4 and CHOP in hippocampus of TC (*n* = 8), NLH (*n* = 8), and LH (*n* = 5) rats.** mRNA levels were determined by qRT-PCR using SyberGreen^®^. Data are the mean ± SEM. Overall group differences in the three groups are as follows: ATF4: *df* = 2,18, *F* = 3.187, *p* = 0.065; CHOP: *df* = 2,18, *F* = 2.407, *p* = 0.12.

The expression levels of ATF4 and CHOP are depicted in **Figure 6**. When the expression level of ATF4 was compared, no significant overall differences were noted between TC, NLH, and LH groups (*df* = 2,18; *F* = 3.18; *p* = 0.065). Further analysis revealed significant upregulation of ATF4 gene in LH group compared with NLH group (*p* = 0.022). When LH group was compared with TC group, no significant differences were noted (*p* = 0.104). There was also no significant difference in ATF4 expression between NLH and TC (*p* = 0.37) groups. Similar to ATF4, there was no significant difference in the expression of CHOP between TC, NLH, and LH groups (*df* = 2,18; *F* = 2.40; *p* = 0.119). The expression of CHOP was significantly higher in the LH group compared with NLH group (*p* = 0.042) but not when compared with the TC group (*p* = 0.209). The expression level of CHOP was not significantly different between NLH and TC groups (*p* = 0.323).

## Discussion

Stress, whether emotional, physical or biological, contributes to disease states and prolonged exposure to stress can lead to severe cognitive impairment and emotional dysfunction as seen in stress-test paradigms and psychiatric illnesses including major depression. For example, stressors have been documented to reduce learning, memory, and cognition in both humans (Tollenaar et al., 2008) and rats (Nestler et al., 2002; Dwivedi, 2009; Zhang et al., 2014). In recent years, there have been several clinical and pre-clinical studies which show that there is a strong connection between depression and UPR (Bown et al., 2000; Zhang et al., 2014). In the present study, we used LH model of depression that has unique advantage in which resiliency (NLH) and vulnerability to develop depression (LH) can be distinguished behaviorally. Studying the UPR differences in these two groups of rats provides opportunity to further understand the mechanisms that can be responsible to develop depression in susceptible individuals.

GRP78 and GRP94 are two critical markers of UPR. In the present study we found that expression of both GRP78 and GRP94 were upregulated in hippocampus of LH rats. Functionally, GRP78, also known as BiP, is responsible for negatively regulating the UPR by interacting with the three sensor proteins, PERK, IRE1, and ATF6 that detect misfolded proteins (Kaufman, 2002). When BiP reacts to an unfolded protein, it releases the sensor protein and allows them to perform their intracellular functions. Thus, elevated level of GRP78 is indicative of ATF6 activity, which transcribes more chaperones in response to misfolded proteins. Under homeostatic conditions, the GRPs are responsible for chaperoning peptides and proteins, Ca^2+^ binding, and cytoprotection (Lee, 2001). GRP94 is responsible for chaperoning the toll-like receptors 2, 4, 5, 7, and 9 which have critical roles in the function of antigen presenting cells (APCs) which produce pro-inflammatory cytokines and prime the adaptive immune response (Yang et al., 2007). Thus, increased expression of GRP94 is indicative of ER-stressed cell priming for inflammatory interactions, which is quite relevant to depression as several studies show increased inflammatory response in depressed patients (Lotrich, 2012; Felger and Lotrich, 2013).

Another connection to the UPR is induction of cytokines and inflammation that have been linked to depression. IL-6, TNFα, IL-1β and their soluble receptors have been found to be upregulated in patients with major depression (Lotrich, 2012; Felger and Lotrich, 2013). CHOP, one of the key regulators of UPR, directly induces caspase 11 which activates IL-1β. The UPR also acts on other pro-inflammatory cytokines such as IL-8 (Vij et al., 2008) and TNFα (Kim et al., 2015) via the IRE-1-XBP-1 pathway, and IL-23 via CHOP (Goodall et al., 2010). As mentioned above, ATF6 is one of the sensor proteins that is responsible for detecting and responding to misfolded proteins in the ER lumen (Kaufman, 2002). It is not surprising that hippocampus of LH rats showed the increased expression of both the chaperones (GRP78 and GRP94) as well as ATF6. The transmembrane protein ATF6 is released from the ER lumen by cleavage when BiP reacts to a misfolded protein. ATF6 is then trafficked to the Golgi apparatus where further cleavage by S1P and S2P proteases occurs. Finally, ATF6 migrates to the nucleus where it activates the transcription of chaperone proteins that restore proper folding in the ER lumen (Ye et al., 2000). Increased expression levels of XBP-1 and CHOP in LH rats indicate their possible role in inflammation and subsequent development of depression. XBP-1 is also the substrate of the BiP-bound ER-stress sensor IRE1. Once released from BiP, IRE1 is homodimerized, and phosphorylated, which then cleaves XBP-1. The active XBBP-1 leaves the ER and is translocated into the nucleus where it begins its transcriptional activity. XBP-1 has been found to constitutively bind to genes involved in ER homeostasis which includes processes like protein folding, trafficking and the ER-associated degradation (ERAD) components (He et al., 2010). In addition, as mentioned above, XBP-1 is vital in signaling cytokine activity like TNF-α (Kim et al., 2015) and is considered to be an important step in determining if the cell will follow the pro-apoptotic path or if the cell will be rescued from the ER-stress (Kaufman, 2002; Ron, 2002). Thus increase in the expression levels of XBP-1 in brain of LH rats may have several implications including apoptosis and increased inflammatory response.

Like XBP-1, ATF4 is a master regulator and a primary determinant of cell survival. Continuous expression of ATF4 can lead to reduced expression of proteins that are pro-survival. ATF4 is regulated by PERK. When released by BiP, PERK dimerizes and is activated which, in turn, phosphorylates eukaryotic initiation factor 2 alpha kinase (eIF2α). This ultimately leads to the preferential transcription and translation of ATF4 mRNA despite reducing overall translation and protein synthesis (Kaufman, 2002; Ron, 2002). ATF4 promotes the activation of the CHOP gene which is responsible for the activation of pro-apoptotic genes and inducing several apoptotic pathways (Harding et al., 2000). CHOP has been shown to induce ERO1α (an oxidase which catalyzes oxidation in the ER lumen) and BIM while simultaneously decreases the expression of BCL-2, which is an anti-apoptotic protein (Tsai and Weissman, 2010). In conjunction with altering the oxidative state of the ER lumen, CHOP expression ultimately enhances protein expression, effectively reversing the protein degradation and synthesis-halting activity of other UPR genes, which overwhelms the protein load of the ER, and forces it to move to apoptosis (Marciniak et al., 2004). Although, we did not find significant change in the expression of ATF4 and CHOP, our data indicate a trend toward their increased expression in LH rats and decreased expression in NLH rats. This raises an interesting possibility of ATF-mediated regulation of CHOP in NLH and LH rats and their role in resiliency vs. vulnerability in developing depression.

As described previously, the IRE-1/XBP-1 pathway are involved in signaling the ERAD system. Once the ERAD is primed for degradation by processes like those of CHOP and ATF4, apoptotic pathways are initiated. IRE-1 activates apoptotic signaling kinase 1 (ASK1) through TNF receptor-associated factor 2 (TRAF2) binding which activates the c-Jun N-terminal kinase and P38-mitogen activated protein kinase pathways. This happens due to CHOP’s downregulation of BCL-2 which protects the cell from the activity of JNK. Similarly, in these conditions, IRE-1 has shown activation of CASP4/Caspase 12 (Tsai and Weissman, 2010). CHOP also has a more active role in transcription and activation of apoptotic signals. Yamaguchi and Wang (2004) showed that CHOP upregulates the pathway that allows for cytokine activity to promote cell death. CHOP upregulates DR5 (death receptor 5), which in turn, acts on BAX and Caspase 3, both of which are well known apoptotic factors. CHOP is also important for the induction of Caspase 11 which is necessary for pro-caspase 1 and IL-1β, a pro-inflammatory cytokine (Endo et al., 2006).

Overall, our study suggests that the cellular response to ER stress, the UPR, is active in the hippocampus of LH rats. This is evident from our findings demonstrating that all three major pathways of UPR system had increased activity in hippocampus of LH rats which indicates the possibility that hippocampal cells are physically stressed under depressive conditions. Our study also suggests that the abnormal UPR system is specifically associated with LH behavior and is not a random response to stress as both LH and NLH rats were subjected to the same stress paradigms. Interestingly, the CORT levels between NLH rats and LH rats were significantly different (higher in LH than NLH rats). Whether differences in CORT levels are responsible for higher UPR in LH rats needs to be further examined. This is an interesting observation which suggests that ER stress is abnormally active only when rats are highly depressed. This needs to be confirmed using animal model systems that show mild or moderate depressive behavior. As we have discussed a number of possibilities how individual components of UPR system can induce cell death or can induce inflammation; our next step would be to delineate such mechanisms and examine if there are morphological changes in brain of LH rats induced by abnormal UPR. We would also like to expand this investigation in other brain regions to examine the specificity of these changes.

## Author Contributions

MT performed experiments, YD supervised the experiments, and MT and YD together wrote the manuscript.

## Conflict of Interest Statement

The authors declare that the research was conducted in the absence of any commercial or financial relationships that could be construed as a potential conflict of interest.
